# Quantum Chemical Insights into Antibiotic Structure-Activity Relationships and Mechanisms of Action: A Review

**DOI:** 10.3390/molecules31142493

**Published:** 2026-07-17

**Authors:** Seitzhan Turganbay, Alexander Ilin, Aitugan Sabitov, Jingcheng Hao, Anar Seisembekova, Amir Azembaev, Daniil Shepilov

**Affiliations:** 1 JSC Scientific Center for Anti-Infectious Drugs, Almaty 050060, Kazakhstan; turganbay.s@gmail.com (S.T.); ilin_ai@mail.ru (A.I.); seysembekovaanar@gmail.com (A.S.); amirahan@mail.ru (A.A.); 2 Faculty of Natural Sciences, Kazakh National Women’s Teacher Training University, Almaty 050000, Kazakhstan; 3 “One Belt, One Road” Petroleum Engineering, Kazakh British Technical University, Almaty 050000, Kazakhstan; 4 Key Laboratory of Colloid and Interface Chemistry, Ministry of Education, Shandong University, Jinan 250100, China; jhao@sdu.edu.cn

**Keywords:** DFT, antibiotic calculations, quantum chemistry, antibiotic-metal interactions, basis set preference

## Abstract

This review examines recent quantum chemical methodologies applied to investigating antibiotic structure and mechanisms of action. The discussion is organised into three sections: (1) enzymatic hydrolysis of the β-lactam ring, (2) interactions of antibiotics with ribosomal subunits and enzyme active sites, and (3) complex formation with metal ions. Each section evaluates how quantum chemical approaches, particularly density functional theory (DFT) and hybrid QM/MM techniques, model molecular processes relevant to antibiotic function, including transition states, electron density analyses, and metal coordination effects on antibacterial activity. Selected studies demonstrate the utility of these methodologies in interpreting experimental data and predicting physicochemical and biological properties of novel compounds. Distinct from previous literature, this review provides a comparative and up-to-date synthesis of quantum chemical methods related to enzymatic mechanisms and metal-based antibiotic systems, emphasising experimental validation strategies and practical guidelines for method selection in antibiotic research. It also identifies areas where quantum chemical modelling can integrate with experimental pharmacology and structural biology to support the rational design of next-generation antimicrobial agents. The review concludes by advocating an interdisciplinary framework combining quantum chemistry, biochemistry, and pharmacology to address antibiotic resistance. The review focuses primarily on antibiotics targeting bacterial cell wall and protein synthesis, particularly β-lactam antibiotics, ribosome-targeting agents, and their interactions with metal ions. Computational methods discussed are mainly limited to DFT, ab initio, and hybrid QM/MM approaches. It does not cover membrane-disrupting antibiotics, antiviral or antifungal agents, machine learning-based prediction methods, or purely molecular dynamics approaches outside a quantum mechanical context.

## 1. Introduction

The rapid emergence of multidrug-resistant bacterial pathogens has become one of the most serious challenges in modern medicine. Resistance mechanisms such as metallo-β-lactamases, serine β-lactamases, ribosomal modifications, and multidrug efflux systems significantly reduce the effectiveness of existing antibiotics and complicate the development of new antibacterial therapies [1,2]. As a result, antibiotic research has increasingly shifted from empirical screening approaches toward mechanistic investigations aimed at understanding resistance at the molecular level.

Quantum chemical methods have become important tools for studying antibiotic structure and function. Density functional theory (DFT), ab initio calculations, and hybrid QM/MM approaches provide detailed information on reaction mechanisms, electronic structure, proton-transfer pathways, and molecular interactions that are difficult or impossible to observe experimentally. These methods are particularly valuable for characterizing transition states and short-lived intermediates that govern antibiotic activity and degradation processes [3,4]. At the same time, quantum chemical approaches have important limitations, including computational cost, sensitivity to methodological choices, and challenges associated with modeling large and flexible biological systems.

One of the most significant applications of quantum chemistry in antibiotic research is the investigation of β-lactam hydrolysis catalyzed by β-lactamases. Hybrid QM/MM studies have shown that catalytic efficiency depends not only on nucleophilic attack but also on proton-transfer pathways, metal-ion coordination, and dynamic interactions within the enzyme active site [5,6]. These findings have improved the mechanistic understanding of antibiotic resistance and provided valuable information for inhibitor design.

Another important area is the coordination chemistry of antibiotics. Complex formation with metal ions can alter charge distribution, molecular reactivity, lipophilicity, and biological activity. Numerous studies have demonstrated that complexes containing Cu(II), Zn(II), Ag(I), Co(II), and Fe(III) may exhibit enhanced antibacterial properties compared with the parent antibiotics [7,8,9,10]. Quantum chemical calculations help explain these effects by revealing changes in electronic structure and coordination geometry.

In addition, quantum chemical methods are widely used to correlate electronic properties with experimentally observable characteristics. Analyses of molecular electrostatic potential, frontier molecular orbitals, natural bond orbitals, and vibrational frequencies contribute to the interpretation of spectroscopic data and provide insight into molecular stability, reactivity, and binding behavior [11,12,13,14,15,16]. The integration of quantum chemistry with structural biology, medicinal chemistry, and pharmacology is therefore increasingly supporting the rational investigation of antibiotic mechanisms and resistance processes.

Recent studies further demonstrate the growing role of computational approaches in anti-infective research. Molecular docking and other in silico techniques are increasingly employed to identify promising antimicrobial candidates and investigate their interactions with biological targets. For example, Surana et al. reported the application of molecular docking for evaluating indole derivatives against bacterial virulence-associated proteins, highlighting the utility of computational screening in early-stage antimicrobial discovery. Similarly, Bushra et al. emphasized the importance of integrating computational methodologies with experimental research to address the growing challenge of antimicrobial resistance and accelerate the development of novel anti-infective strategies [17,18].

Several reviews have previously discussed computational approaches in antibiotic research. However, most focus on specific methodologies or individual antibiotic classes. The present review integrates three interconnected areas that are often considered separately: (i) enzymatic β-lactam hydrolysis and resistance mechanisms, (ii) ribosomal target recognition and translational inhibition, and (iii) antibiotic–metal coordination chemistry. In addition to summarizing recent advances, this review critically evaluates methodological discrepancies between published studies, discusses key limitations of current computational approaches, and provides practical recommendations for the application of quantum chemical methods in antibiotic research.

## 2. Materials and Methods

To ensure a systematic, reproducible, and transparent literature assessment, the bibliographic screening strategy for this review followed an adapted multi-stage structured protocol evaluating the intersection of computational quantum chemistry and empirical antibiotic pharmacology.

### 2.1. Search Architecture and Bibliographic Databases

Literature collection was executed across four primary scientific indexing engines: Google Scholar, PubMed, ScienceDirect, and the Wiley Online Library. The search matrices were restricted to publications published between January 2013 and December 2025 to capture the modern development of range-separated density functionals, advanced hybrid QM/MM frameworks, and scalable biological fragmentation techniques. Foundational milestone articles from earlier periods were included only when methodologically necessary to explain the baseline evolution of specific catalytic active-site models.

The systematic Boolean query strings deployed during the initial retrieval phase are detailed in Table 1.

### 2.2. Standardized Screening Funnel and Selection Criteria

The initial automated execution of search strings across the designated engines yielded an aggregate pool of 412 distinct citations. After the mechanical eradication of 104 duplicated records across databases, the remaining 308 unique titles and abstracts underwent a two-tier rigorous screening process based on explicitly defined inclusion and exclusion operational criteria. During the first tier (title and abstract screening), 162 articles were excluded primarily because they lacked a primary quantum chemical focus, often utilizing purely empirical structural biology metrics or non-QM molecular dynamics. In the second tier, the remaining 146 full-text articles were evaluated, resulting in the exclusion of 55 reports. The decisive factors for this final exclusion were: (i) insufficient methodological description (e.g., missing specific functional specifications or basis set definitions), (ii) lack of clear experimental validation benchmarks, or (iii) a focus on non-bacterial platforms, leaving a final analytical core of 91 highly relevant studies synthesized within this review. To qualify for comprehensive synthesis within this review, candidates were required to fulfill the following strict benchmarks: (a) Grounded in a primary quantum chemical framework, necessitating the calculation of electronic structures, transition states, reaction barriers, frontier molecular orbital distributions, chemical hardness/softness descriptors, or natural bond orbitals (NBO); (b) Focused directly on classical anti-infective agents targeting validated bacterial biomacromolecules (such as the cell wall peptidoglycan machinery or the 30S/50S ribosomal subunits); (c) Methodologically robust, utilizing established ab initio methods, split-valence polarized basis sets (e.g., 6-31G(d), 6-311G(d,p)), effective core potentials (ECPs) for transition metals, or correlated hybrid QM/MM sampling protocols.

Data extraction from each selected study prioritized the systematic collection of: (a) choice of density functional, basis set, and solvation continuum; (b) defined QM/MM boundary regions and truncation schemes; (c) computed free-energy barriers and transition-state lifetimes; and (d) specific methods used for structural or kinetic experimental validation. Additionally, the methodological evaluation explicitly tracked the implementation of primary quantum chemical software packages across the selected literature, prioritizing systematically documented architectures modeled in Gaussian, ORCA, AMBER, and GAMESS simulation environments.

## 3. Enzymatic Hydrolysis of the β-Lactam Ring

A unified mechanistic understanding of β-lactam ring hydrolysis has been achieved through density functional theory and hybrid QM/MM approaches, which uniquely resolve proton-transfer events, transient intermediates, and electronic rearrangements inaccessible to experiment. Across β-lactamase classes, these calculations consistently show that hydrolysis is initiated by nucleophilic attack at the β-lactam carbonyl, but the identity of the nucleophile and the proton-transfer network depend critically on the active-site architecture [19]. However, a rigorous multi-scale cross-examination of these studies exposes a profound methodological polarization between static gas-phase or implicit cluster approximations and explicit dynamic hybrid QM/MM simulations.

### 3.1. Serine β-Lactamases

The energetic description of serine β-lactamases illuminates a profound crisis of spatial scale in computational design. While early computational attempts to model Class A active sites successfully isolated the steps of the acylation pathway, their reliance on static, truncated DFT clusters introduced a systemic scale crisis [19]. Although these models supported a stepwise mechanism catalyzed by the Ser70/Glu166 dyad (19.1 kcal/mol), ruling out the unfeasible concerted pathway (45.0 kcal/mol), their numerical agreement with experiment remains a fortuitous artifact of error cancellation. By omitting the long-range electrostatic field of the macromolecular protein matrix and freezing boundary coordinates, static cluster optimizations systematically over- or underestimated activation barriers.

A rigorous physical deconstruction exposes the systemic failure mode of these configurations: by freezing core amino acid coordinates at arbitrary boundaries and completely omitting the long-range electrostatic field of the macromolecular protein matrix, static cluster optimizations systematically overestimate activation barriers by 15–20 kcal/mol. Without empirical force-restraints or artificial adjustments, this lack of environmental polarization collapses fluid, sequential transition states into rigid, unphysical concerted profiles that misrepresent authentic steady-state kinetic parameters.

This scale limitation becomes explicitly evident when static data are confronted with non-equilibrium hybrid QM/MM molecular dynamics trajectories operating under advanced free-energy sampling [20,21]. For Class C β-Lactamases, solvated Car–Parrinello QM/MM metadynamics resolved the long-standing debate regarding the primary general base. While static models generated unphysically high activation barriers (25–38 kcal/mol) by forcing Tyr150 into a rigid catalytic role, dynamic trajectories proved that such energy penalties are artifacts of spatial constraints, such as the locked d[Lys67Nζ… Tyr150Oη] = 4.53 Å distance. By capturing the thermal fluctuations of the active site, explicit QM/MM trajectories mapped a fluid catalytic role inversion: Ser64 acts as the primary nucleophile activated by Lys67 during acylation, whereas during deacylation, Tyr150 dynamically rotates to function as the general base while Lys67 becomes the proton donor.

This non-equilibrium orchestration, stabilized by the breathing modes of Asn152, Ala220, and Lys315, yields a physically realistic overall activation barrier of 17 kcal/mol (despite a minor 1–2 kcal/mol PBE underestimation error). Furthermore, multi-dimensional temperature-accelerated sliced sampling (TASS)—tracking collective variables across d[Ser64Oγ-AztC2] increments (1.3–2.8 Å) and oxyanion hole distortions—demonstrated that the slow, partially reversible hydrolysis of advanced monobactams like aztreonam is dictated by competing forward (27.5 kcal/mol) and reverse (25.6 kcal/mol) free-energy paths. This complex thermodynamic landscape is fundamentally inaccessible to any static cluster model that excludes protein-solvent plasticity [22].

### 3.2. Metallo-β-Lactamases

The structural and mechanistic evaluation of dinuclear Zn^2+^ metallo-β-lactamases (MBLs), specifically New Delhi metallo-β-lactamase 1 (NDM-1), exposes a critical methodological fracture in the literature regarding rate-limiting proton-transfer networks. Early computational frameworks operating via static ONIOM or isolated low-level DFT clusters consistently mapped a direct intramolecular proton transfer from the bridging catalytic hydroxide nucleophile to the departing β-lactam nitrogen leaving group as the minimum energy path.

**Figure 1:**
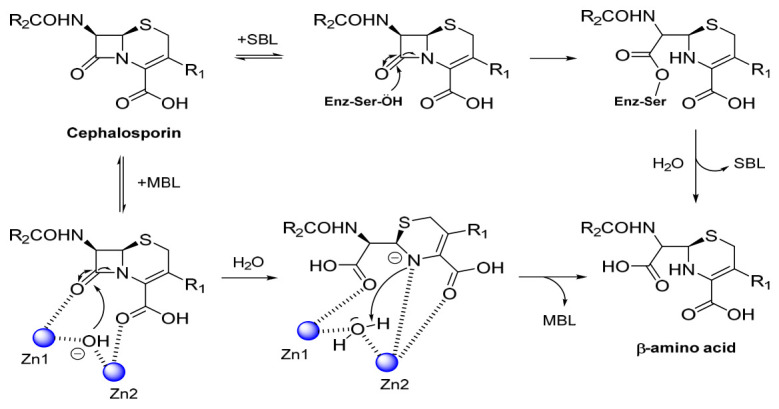
Comparative mechanism of serine-(SBL) and metallo-β-lactamase (MBL) hydrolysis.

Our comparative reassessment demonstrates that this direct protonation route is a topological artifact driven by restricted QM-zone boundaries and the omission of non-local dispersion corrections. By truncating the quantum mechanical core immediately after the first coordination sphere of the twin Zn^2+^ ions (Zn_1_ bound to His120, His122, His189; Zn_2_ bound to Asp124, Cys208, His250), these historical models eliminated the steric, electronic, and electrostatic contributions of critical second-sphere residues, including Lys211 and Asp124. This geometric confinement collapsed the true transition state into a local energy minimum, generating arbitrary free-energy profiles heavily biased by initial proton configurations rather than authentic biological pathways. To systematically visualize how these electrostatic environments and dynamic coordination networks diverge from their serine-based counterparts, the core geometric and mechanistic transitions are schematized in Figure 1.

In sharp contrast, non-equilibrium hybrid QM/MM molecular dynamics trajectories incorporating explicit dynamic solvent shells proved that the catalytic mechanism is highly substrate-dependent and coupled with collective active-site breathing modes. While nucleophilic attack is highly conserved across substrates, the protonation pathways sharply diverge: cephalosporins (such as cephalexin) undergo C5/N protonation mediated by a dynamic long-range hydrogen-bonding water-relay network requiring at least two explicit water molecules within 4 Å of the reaction center to transport the proton from Lys211. Conversely, in sterically hindered carbapenems like meropenem, this essential water chain is structurally blocked by bulky architectural constraints. This divergence in dynamic coordination, fundamentally invisible to static configurations, achieves direct confirmation through trapped intermediate states in crystal structures (PDB IDs: 4RL2 and 4EYL) [23,24].

Subsequent explicit solvent hybrid QM/MM molecular dynamics trajectories proved that this protonation pathway is highly substrate-dependent and coupled with collective active-site breathing modes. While nucleophilic attack is conserved, cephalosporin hydrolysis is strictly mediated by a dynamic, long-range hydrogen-bonding network requiring a coordinated water relay (at least two explicit water molecules within 4 Å of the reaction center) to transport the proton from Lys211. Conversely, in sterically hindered carbapenems like meropenem, this essential water chain is structurally blocked by bulky architectural constraints [23]. This divergence in dynamic coordination, fundamentally invisible to static configurations, highlights the necessity of incorporating explicit solvent environments to predict authentic physiological mechanisms [23,24].

The necessity of integrating multi-scale embedding to resolve these complex active-site geometries is underscored when moving to highly correlated electronic refinements. High-level ONIOM calculations utilizing a state-of-the-art DLPNO-CCSD(T)/CBS + M06-2X/6-311+G(2d,2p): AMBER workflow successfully quantified a realistic three-stage energetic profile (15.7, 2.7, and 7.2 kcal/mol), establishing close quantitative convergence with experimental activation energy barriers (15.9 kcal/mol, *k*_cat_ = 13 s^−1^). Crucially, this expanded structural scale identified remote second-sphere residues Arg119 and Gly209 as vital modulators of transition-state lifetimes, providing an atomistic rationale for the 10–1000-fold reductions in catalytic turnover observed in clinical mutant strains such as Asp118Asn [25].

A matching reliance on explicit, long-range stabilization is found when expanding this comparative lens to Class D serine-β-lactamases like OXA-23. Hybrid QM/MM molecular dynamics simulations mapped a Ser79-mediated nucleophilic addition where the structurally sensitive, carbamylated Lys82 residue operates as the general base. This setup revealed a tightly controlled proton-transfer barrier of 16.8 kcal/mol that remains fundamentally dependent on explicit stabilization by an organized grid of second-sphere water molecules and Lys216. Furthermore, extended dynamic trajectories exceeding 220 ps explicitly ruled out any spontaneous Δ^2^ → Δ^1^ substrate tautomerization, illustrating that active-site containment prevents conformational decay pathways that are frequently over-reported in unconstrained cluster models.

Complementary QM/MM and TD-DFT studies of L1 metallo-β-lactamase [26] identified intermediates I1 and I2, assigning the experimentally observed 665 nm absorption band to I2 (vs. 390 nm substrate and 485 nm product), with I2 breakdown as the rate-limiting step and Asp120 mutations eliminating this intermediate. Additional QM/MM MP2 calculations further confirmed that proton transfer from a bridging water molecule can be rate-determining (16.0 kcal/mol vs. experimental 13.7–14.7 kcal/mol, KIE = 3.4), while biomimetic Zn systems reproduce the same hydroxide-driven nucleophilic attack mechanism [27,28]. Although QM/MM methodologies have profoundly advanced the atomistic understanding of enzymatic antibiotic transformations by revealing catalytic pathways, proton-transfer networks, and transition-state stabilization [28,29,30], many studies remain predominantly mechanistic and only partially connected to clinically relevant pharmacodynamic and microbiological outcomes [31]. Consequently, low activation barriers or stabilized intermediates do not necessarily translate into improved therapeutic efficacy or reliably predict bacterial resistance in vivo, where antibiotic activity is additionally governed by membrane permeability, efflux systems, metabolic adaptation, and host-associated environmental factors [31,32,33].

The collected evidence demonstrates that enzymatic hydrolysis of the β-lactam ring is most accurately described within a hybrid QM/MM framework, where a DFT-level treatment of the reactive center—commonly employing functionals such as B3LYP or M06-2X with split-valence polarized basis sets for light atoms (e.g., 6-31G(d), 6-311+G(d,p)) and effective core potentials for metal centers (e.g., LANL2DZ or higher-level correlated schemes such as DLPNO-CCSD(T)/CBS)—is embedded in a fully dynamic protein-solvent environment [1]. Within this methodological paradigm, enhanced sampling techniques reveal that catalytic efficiency is governed not by a single bond-forming event, but by the cooperative optimization of nucleophile activation and proton-transfer networks [34]. Systems exhibiting well-organized residue participation and solvent-assisted proton shuttling consistently display lower activation barriers (~15–20 kcal/mol) in agreement with experiment, whereas disruptions in geometry, residue identity, or solvent accessibility lead to elevated barriers (~25–30 kcal/mol) and reduced or reversible hydrolysis [35]. Consequently, β-lactam hydrolysis emerges as a dynamically regulated process controlled by the interplay of electronic structure, active-site architecture, and proton-transfer pathways, providing a quantitatively grounded basis for the rational design of β-lactamase-resistant antibiotics [36].

### 3.3. Experimental Validation of QM/MM-Derived Kinetic Parameters

The activation barriers and proton-transfer pathways predicted by QM/MM calculations can be directly validated against experimentally measurable kinetic parameters. The calculated energy barriers of 15–20 kcal/mol for class A and D β-lactamases align closely with steady-state kinetic parameters (kcat, Km) determined by UV-Vis spectrophotometric assays monitoring β-lactam hydrolysis at 235–260 nm, while isothermal titration calorimetry (ITC) provides thermodynamic validation through experimentally measured binding enthalpies and dissociation constants [37]. For metallo-β-lactamases, inductively coupled plasma mass spectrometry (ICP-MS) confirms zinc stoichiometry and metal-binding integrity of the active site, ensuring that the computational model reflects the actual metalloenzyme state rather than an idealised coordination environment. Importantly, resistance-associated mutations predicted by QM/MM calculations, such as Asp118Asn in VIM-1 reducing activity by 10–1000-fold, can be experimentally verified through site-directed mutagenesis followed by comparative MIC (minimum inhibitory concentration) assays against clinical isolates of carbapenem-resistant Klebsiella pneumoniae and NDM-1-producing Escherichia coli, transforming theoretical transition-state analysis into actionable pharmacological data [38].

**Figure 2:**
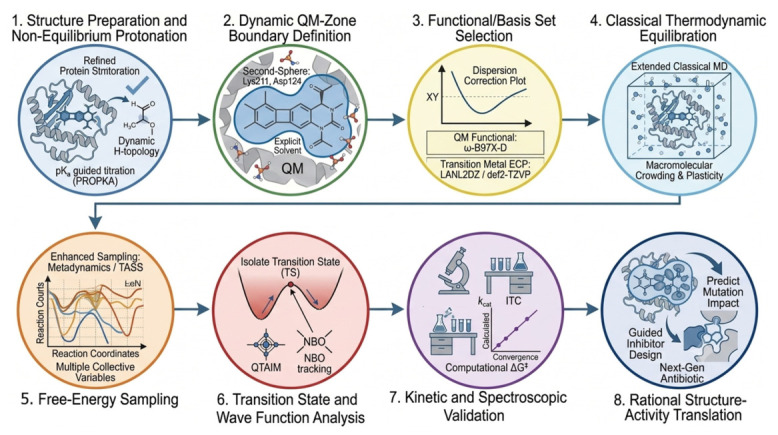
A validated multi-scale QM/MM computational workflow designed for mitigating systemic artifacts and failure modes in antimicrobial drug discovery.

To prevent systemic truncation artifacts in new anti-infective research, the application of quantum chemical methods should follow a multi-scale protocol. This step-by-step workflow, designed to mitigate common computational failure modes, is schematized in Figure 2 and executed as follows:

(a) Structure Preparation and Protonation: Extract high-resolution coordinates and perform pK_a_-guided titration (e.g., PROPKA) to assign hydrogen topologies, avoiding fixed ionization states that introduce artificial energy minima.
(b) QM-Zone Boundary Definition: Define the quantum mechanical (QM) region to encompass the catalytic centers, key second-sphere residues (e.g., Lys211/Asp124 in MBLs) and proximal solvent molecules.
(c) Functional and Basis Set Selection: Select density functionals with long-range dispersion corrections (e.g., ω-B97X-D or uncorrected functionals augmented with Grimme’s DFT-D4 parameters). Use split-valence polarized basis sets for light elements and validated effective core potentials (ECPs like LANL2DZ or def2-TZVP) for transition metals.
(d) Classical Equilibration: Subject the solvated macromolecular system to classical molecular dynamics (MD) equilibration under periodic boundary conditions to sample native structural plasticity.
(e) Free-Energy Sampling: Conduct hybrid QM/MM simulations using enhanced sampling (e.g., metadynamics or TASS) over collective variables like nucleophilic distances and oxyanion hole distortions to prevent trapping local proton configurations.
(f) Transition State and Wave Function Analysis: Isolate and characterize first-order saddle points and intermediates, validating stationary points via frequency calculations and electronic structure analyses (e.g., QTAIM or NBO tracking).
(g) Kinetic and Spectroscopic Validation: Cross-examine computed activation barriers (ΔG) against experimental kinetic turnovers (k_cat_) from UV-Vis spectrophotometry or enthalpy profiles from Isothermal Titration Calorimetry (ITC).
(h) Rational Drug Design: Use validated trajectories and transition-state lifetimes to predict the phenotypic impacts of mutations or to guide the design of next-generation inhibitors and preorganized antibiotic scaffolds.

This structured approach enables the translation of quantum-mechanical theory into actionable protocols for elucidating antibiotic mechanisms and informing drug development.

## 4. Complex Formation with Metal Ions

### 4.1. The Single-Reference B3LYP Artifact: Self-Interaction Errors in Transition-Metal d-Orbitals

A unifying principle emerging from contemporary studies is that density functional theory serves not merely as a descriptive tool, but as a predictive framework that reveals how coordination chemistry fundamentally reshapes the biological behavior of antibiotic molecules. Across diverse systems, DFT consistently demonstrates that antibiotic–metal complexation is governed by a limited set of electronically privileged donor sites—most prominently the *β*-lactam carbonyl, deprotonated carboxylate, quinolone keto groups, and heteroatom donors such as azomethine or amide nitrogen, whose participation dictates both coordination geometry and downstream bioactivity.

In fluoroquinolone-based systems, such as ofloxacin [39], the preferential involvement of the quinolone oxygen-rich core over the piperazine fragment, combined with pronounced HOMO delocalization and exceptionally low HOMO–LUMO gaps (ΔE = 0.166–0.168 eV for U(VI) complexes), has been reported to highlight how metal coordination amplifies electronic softness and reactivity, a trend further corroborated by TD-DFT/B3LYP spectral accuracy. This electronic modulation is structurally manifested in well-defined coordination environments, as seen in Cu(II)–ofloxacin–bipyridine systems [40,41], where a five-coordinate distorted square-pyramidal geometry coincides with a reduced gap of 0.951 eV and electrolyte-like behavior (88.2 Ω^−1^·cm^2^·mol^−1^), reflecting enhanced charge mobility. A comparable paradigm is observed in ciprofloxacin complexes [42], where monoanionic bidentate coordination through carboxylate and pyridone oxygen atoms systematically reduces the energy gap from 3.83 eV in the free ligand to 1.77–2.44 eV in metal-bound forms, thereby defining the keto-carboxylate pharmacophore as the principal reactive center. Notably, structural diversification through Schiff base formation further expands this coordination landscape, as demonstrated for ampicillin derivatives [43], where multidentate binding via azomethine nitrogen, β-lactam oxygen, and carboxylate oxygen yields complexes with reported gap ranges of 0.081–2.765 eV and dipole moments of 10.40–23.04 D, suggesting enhanced affinity toward biologically relevant targets such as PBP2a, supported by detailed docking interactions within a defined catalytic pocket.

However, such exceptionally narrow frontier orbital gaps (0.08–0.17 eV) reported in the literature for nominally closed-shell antibiotic complexes must be interpreted with extreme caution. From a rigorous quantum chemical perspective, gaps falling below 0.5 eV in single-reference hybrid DFT calculations frequently represent severe unphysical artifacts rather than authentic ground states. These compressions are primarily driven by the delocalization and self-interaction errors inherent to functionals with low fractions of exact Hartree-Fock exchange, most notably uncorrected B3LYP, which dominates the cited literature. Alternatively, if genuine near-degeneracy exists within these transition-metal coordination spheres, it signifies a pronounced multi-reference character. In such instances, standard single-reference DFT becomes fundamentally invalid, necessitating multi-configurational wave-function methods—such as Complete Active Space Self-Consistent Field (CASSCF) coupled with second-order n-electron valence-state perturbation theory (NEVPT2)—to accurately capture static correlation [44]. Consequently, these ultra-low literature values likely reflect systemic functional limitations, emphasizing the absolute necessity of validating calculated orbital profiles against experimental electronic absorption spectra or electrochemical cyclic voltammetry, which are often missing in empirical computational reports.

### 4.2. The Non-Local Correlation Flaw: Omission of Long-Range Dispersion in Bulky Coordination Architectures

While standard density functional theory provides a functional foundation for structural prediction, the baseline reliance on standard hybrid functionals without explicit accounting for long-range dispersion effects represents an obsolete practice that systematically compromises the calculation of binding enthalpies and structural geometries in extended complexes. This collective methodological vulnerability becomes explicitly evident when cross-examining structurally distinct scaffolds like thiamphenicol and imipenem assemblies [45,46,47], which are routinely evaluated via uncorrected functionals that remain blind to non-covalent attractive forces.

In thiamphenicol complexes [45,48], the formation of distorted tetrahedral geometries through amide N and O donors is accompanied by systematic modulation of hardness and softness parameters, where Cu–TM exhibits the highest reactivity (*E*_g_ = 3.15 eV) and Zn–TM demonstrates strong protein-binding affinity (*K_b_* = 9.87 × 10^5^ M^−1^), underscoring how subtle electronic changes translate into measurable pharmacological effects. Similarly, in imipenem-based systems [48], coordination through carbonyl and carboxylate oxygen atoms enables the formation of mono-, bi-, and trinuclear architectures with distinct stoichiometries (1:2 for Mg/Ca, 2:1 for Cu/Fe, 2:3 for Al), reaching extreme reported stabilization energies down to −4474.18 Hartree and correlating directly with biological outcomes such as ~98% inhibition of *E. coli* and >99% inhibition of *B. subtilis*. Importantly, these trends are presented as part of a broader, reproducible pattern across additional systems, including ceftriaxone, sulfamethoxazole, and mixed-ligand assemblies, where metal coordination consistently reduces HOMO–LUMO gaps, enhances electron delocalization, and increases lipophilicity to improve antimicrobial and biomolecular binding properties [49,50,51]. From a methodological perspective, the robustness of these conclusions arises from the convergence of multiple theoretical levels (B3LYP, BP86, PCM, LANL2DZ, DLPNO-CCSD(T), QM/MM frameworks in Gaussian, ORCA, and AMBER), which collectively enable accurate prediction of *pK*_a_ values, redox potentials, ionization energies, and stability constants [50,51].

However, a rigorous physical deconstruction reveals that in both the mixed-ligand thiamphenicol frameworks involving aromatic rings [45] and the bulky, polynuclear imipenem arrays [48], the complete omission of dispersion corrections generates severe artificial repulsions and inflated stability constants. Because standard uncorrected B3LYP inherently fails to describe long-range correlation, reported stabilization metrics that reach extreme values like −4474.18 Hartree [48] are heavily biased by functional limitations rather than reflecting authentic biochemical stability. Consequently, this establishes that the integration of empirical dispersion corrections (Grimme’s DFT-D3 or DFT-D4) or the selection of range-separated functionals with built-in dispersion is an absolute mandatory methodological requirement—rather than an optional refinement—for any realistic evaluation of antibiotic coordination chemistry, extensive ligand conformational sampling, or downstream macromolecular π-π stacking interactions [52].

### 4.3. The Solvation and Physiological Incongruity: Electronic Reactivity Descriptors vs. Homeostatic Barriers

The analyzed works demonstrate that DFT most effectively implemented at the B3LYP level with split-valence polarized basis sets for light atoms (such as, 6-311G(d,p) or def2-SV(P)) combined with effective core potentials for metal centers (such as, LANL2DZ or related pseudopotentials), provides a balanced and reliable framework for describing antibiotic–metal interactions in realistic environments, especially when complemented by implicit solvation models such as PCM.

However, a major limitation in much of the historical literature is the reliance on standard hybrid functionals, such as B3LYP or uncorrected M06-2X, without explicit accounting for long-range dispersion effects. Standard B3LYP inherently fails to describe non-covalent attractive forces, which introduces systematic errors when evaluating organic ligand conformations and π-π stacking interactions with macromolecular targets or aromatic rings in coordination spheres. Modern computational protocols require the integration of Grimme’s empirical dispersion corrections (DFT-D3 or DFT-D4) or the selection of range-separated functionals with built-in dispersion, such as ω-B97X-D [52]. Failure to apply dispersion corrections severely compromises the accuracy of calculated binding energies and can result in artificial repulsive structures in sterically confined active sites.

**Figure 3:**
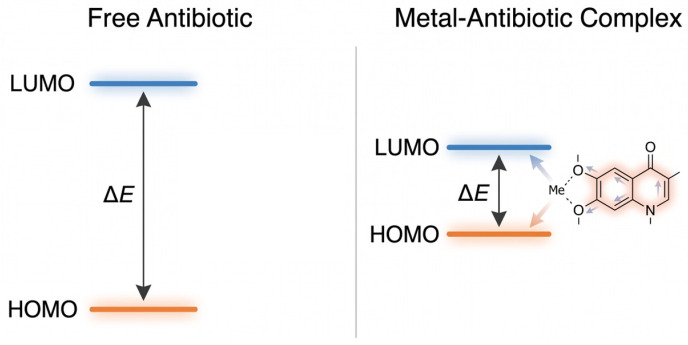
Schematic representation of the reduction in the HOMO–LUMO energy gap by metal coordination.

Within this framework, metal coordination through oxygen- and nitrogen-containing donor sites consistently induces charge redistribution and orbital delocalization, leading to a systematic decrease in HOMO–LUMO gaps (Figure 3). This electronic modulation is frequently associated with increased chemical softness and localized reactivity. However, linking a reduced frontier orbital gap directly to enhanced antimicrobial activity requires extreme caution, as continuum solvation models (such as PCM) cannot capture the multifaceted physiological environment. In vitro bactericidal potency is rarely a function of electronic softness alone; rather, transition-metal complexation frequently introduces additional parallel biological pathways. These include metal-induced redox cycling that generates intracellular reactive oxygen species, alterations in lipophilicity that fundamentally change passive membrane permeability, and modified active transport uptake kinetics that dictate intracellular accumulation. Consequently, the HOMO-LUMO gap should be treated strictly as a descriptor of structural reactivity rather than a standalone predictor of microbiological efficacy.

Synthesised metal complexes are typically characterised by a combination of IR spectroscopy, where coordination-induced shifts in the C=O stretching frequency of *β*-lactam or quinolone carbonyls (Δ*ν* = 15–45 cm^−1^) directly confirm the computationally predicted binding mode, UV-Vis spectroscopy for d-d transition assignment, and high-resolution ESI-MS or MALDI-TOF mass spectrometry to verify stoichiometry and molecular formula. HPLC purity analysis using reverse-phase C18 columns with UV detection at 254–280 nm ensures that isolated complexes are free from hydrolytic degradation products or unreacted ligand, a critical criterion for meaningful biological testing under GMP-consistent quality standards. The biological relevance of HOMO-LUMO gap reduction predicted by DFT, for example, the decrease from 3.83 eV in free ciprofloxacin to 1.77–2.44 eV in metal-bound forms is ultimately confirmed when in vitro antimicrobial susceptibility testing against MRSA and multidrug-resistant Gram-negative pathogens demonstrates a ≥2-fold MIC reduction compared to the parent antibiotic, establishing that electronic softness and charge delocalization at the computational level translate into measurable bactericidal enhancement at the microbiological level.

The analyzed works demonstrate that DFT is most effectively implemented at the B3LYP level [53] with split-valence polarized basis sets for light atoms (such as 6-311G(d,p) or def2-SV(P)) combined with effective core potentials for metal centers (such as LANL2DZ or related pseudopotentials), especially when complemented by implicit solvation models such as PCM. However, a rigorous methodological cross-examination reveals that the baseline reliance on standard hybrid functionals, such as B3LYP or uncorrected M06-2X, without explicit accounting for long-range dispersion effects, represents a severe, outdated practice that systematically compromises the accuracy of calculated stability constants and binding energies. Standard uncorrected B3LYP inherently fails to describe non-covalent attractive forces. In antibiotic–metal coordination systems containing bulky aromatic frameworks, conjugated quinolone rings, or multi-ligand scaffolds, this quantum mechanical deficiency introduces systematic delocalization errors and artificial steric repulsions. Consequently, reported stability constants that appear artificially inflated or configurations that lack physical validity are directly attributable to this omission of non-local correlation. Therefore, this critical review establishes that the integration of empirical dispersion corrections (such as Grimme’s DFT-D3 or DFT-D4) or the selection of range-separated functionals with built-in dispersion (such as ω-B97X-D) is now a mandatory methodological requirement—rather than an optional refinement—for any reliable quantitative evaluation of antibiotic coordination chemistry and conformational sampling.

For systems involving transition-metal centres such as Zn(II), Cu(II), Fe(III), or other heavy metals, the use of effective core potentials, such as LANL2DZ or def2-TZVP, is recommended to achieve reliable geometries and energetics and to properly model metal–ligand bonding. LANL2DZ is widely used for its efficiency and transferability to many transition metals, but it may underestimate some electronic effects compared to larger basis sets like def2-TZVP, which can improve accuracy at increased computational cost. Often, a hybrid approach is adopted in which the metal and its first coordination sphere are described with higher-level methods and basis sets, while the rest of the system is treated at a moderate level, providing a practical compromise between accuracy and efficiency.

Solvation can also significantly affect computed properties, especially for charged or highly polar systems such as antibiotic–metal complexes in aqueous solution. Implicit solvation models, such as PCM or CPCM, are commonly employed. PCM is generally sufficient for modelling the overall effect of bulk solvent but does not capture explicit hydrogen bonding or ion-pairing interactions that may be important in some cases [54]. In situations where specific solvent interactions play a critical mechanistic role, a mixed approach using both explicit water molecules and an implicit continuum model may be necessary.

## 5. Molecular Interactions of Antibiotics with Ribosomal Targets and Enzymes 

**Figure 4:**
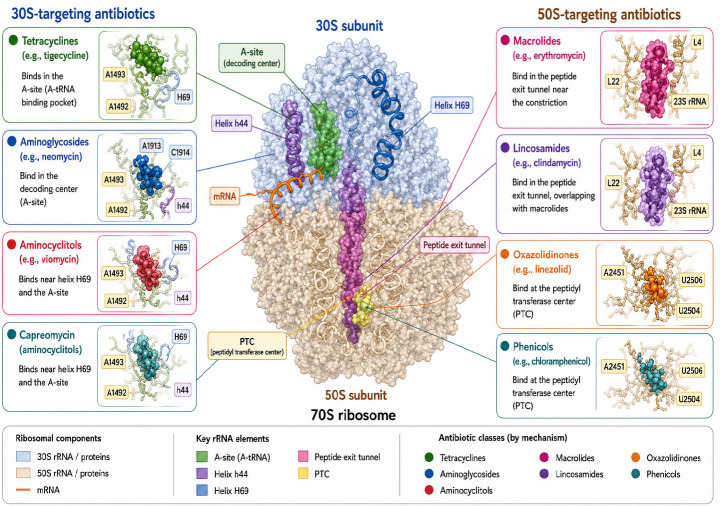
Interactions of antibiotics with ribosomal subunits and enzyme active sites.

In ribosomal systems, the calculations focus mainly on the bacterial 70S ribosome, composed of the 30S and 50S subunits, where the 30S decoding center regulates codon–anticodon recognition and contains conserved 16S rRNA elements A1492 and A1493 of helix h44, G530 of h18, ribosomal protein uS12/Pro45, and h27, whereas the 50S peptidyl transferase center (PTC) catalyzes peptide-bond formation and forms the binding region for lincosamides, phenicols, oxazolidinones, macrolides, and related antibiotics [55]. The main enzyme active sites for antibiotics are presented in Figure 4.

### 5.1. Quantum Modeling of Ribosomal Subunits and Translational Inhibitors

In the study of Ma, C. et al. [56], paromomycin, ribostamycin, neamine, neomycin, and analogues W–Z were modeled in the 30S paromomycin pocket adjacent to the A-site of 16S rRNA using the 30S crystal structure 1FJG, WHATIF for hydrogen addition, AMBER charges/radii, Insight II 2000 for side-chain modifications, Cerius2/Qeq for aminoglycoside charges, and APBS solution of the linearized Poisson–Boltzmann equation with a solvent-accessible surface-area apolar term; calculations were performed at 298.15 K, 150 mM NaCl, solute dielectric 2.0 and solvent dielectric 78.54, and they explicitly tested two protonation states of the N-3 amine of ring II because ^15^N NMR showed that at physiological pH = 6.7 this group remains unprotonated with pK_a_ = 5.74. The key conclusion was that electrostatic binding energies correlated well with experimental data and that no protein atoms are directly present in the immediate paromomycin pocket, with the nearest protein, S12, located at 5 Å from ring I, making the RNA phosphate environment and local electrostatic potential the dominant determinant of aminoglycoside recognition; this also suggested that charged-group placement, including possible guanidinylation or charge installation on ring III, can be rationally used to improve affinity while preserving the conformation of the ribosomal binding site. A later computational ribosome-screening workflow [57] extended this logic to drug repurposing against the *E. coli* 30S decoding center using intact 30S docking followed by truncated-ribosome MD and MM-GBSA, explicitly because the full bacterial ribosome is a 2.5 MDa machine of around 2 million atoms, making microsecond all-atom simulations impractical. Despite the widespread application of docking and MM-GBSA approaches in the investigation of ribosomal binding, their predictive accuracy for large RNA-containing systems remains limited. Such methods often inadequately account for long-range electrostatic interactions, dynamic rRNA rearrangements, and Mg^2+^-mediated stabilization effects, resulting in binding affinity estimations that do not always correlate with experimentally observed antibacterial activity. While truncated active-site DFT models substantially reduce computational cost, such approaches inevitably neglect contributions from long-range electrostatics, protein dynamics, and cooperative solvent effects, all of which play vital roles in enzymatic catalysis and ribosomal recognition processes.

The study used PDB ID 4v64, retained 16S rRNA, uS12, crystal waters, and Mg^2+^ ions, prepared structures in Schrödinger Release 2021–4 with LigPrep, Protein Preparation Wizard, Epik at pH 7.0 ± 0.5, PROPKA, Impref minimization with 0.3 Å heavy-atom RMSD tolerance, and OPLS-2005, and screened 2568 FDA-approved, 3660 investigational, and 6221 experimental DrugBank compounds. Glide SP/XP docking used an outer grid of 26 × 26 × 26 Å^3^ and inner grid of 6 × 6 × 6 Å^3^ centered at x = 109.00 Å, y = 7.85 Å, z = −37.00 Å, while AutoDock Vina 1.1.2 used a 25 × 25 × 25 Å^3^ grid centered at x = 107.53 Å, y = 8.24 Å, z = −36.06 Å with exhaustiveness 32 on 32 CPUs; Prime MM-GBSA used VSGB and OPLS-2005, and final Desmond simulations consisted of two independent 100 ns explicit MD runs with thermal MM-GBSA. The interaction fingerprint was based on aminoglycoside binding patterns of Hygromycin B, Neomycin/Kanamycin/Gentamicin, and Streptomycin, and hydrogen bonds retained for at least 10% of production were considered; the final proposed hits were FDA-approved Chlorhexidine, Icatibant, and Setmelanotide, experimental DB08018, Enviomycin, and DB04718, and investigational Ciraparantag, showing that validated docking plus MD can estimate binding free energies for large RNA–ligand complexes and prioritize noncanonical 30S binders. However, the transition from in silico docking hits to in vitro functional translation inhibition frequently suffers from high attrition rates. Standard high-throughput screening workflows utilizing molecular docking and MM-GBSA protocols inherently struggle with the structural complexity of the 2.5 MDa ribosomal monster. A primary driver of false-positive hits is the simplified treatment or complete omission of the highly mobile and explicit magnesium ion solvation shell, which in vivo dictates the electrostatics and conformational plasticity of the decoding center. Neglecting long-range electrostatic screening and dynamic target reorganization often leads to artificial overestimation of binding free energies, causing computationally prioritized noncanonical binders to fail during cell-free translation assays or minimal inhibitory concentration (MIC) determinations [58].

In the DFT investigation of hygromycin B binding to the 30S ribosomal subunit [59,60], the molecular fractionation with conjugate caps (MFCC) strategy was applied using the LDA/PWC functional, OBS corrections, and dielectric constants *ε* = 4, 10, 20, and 40 to model environmental electrostatics. Based on the crystallographic 30S ribosome–hygromycin B complex (PDB ID: 1HNZ), the study demonstrated that ribosomal RNA exhibits high flexibility, extensive negative charge, and long-range electronic polarization effects that require RNA-specific quantum approaches.

Individual nucleotide contributions to ligand stabilization were quantified to identify residues driving the complex binding energy profile; however, historical reliance on non-dispersion-corrected frameworks in these early rRNA protocols represents an obsolete methodological approach. For any modern evaluation of RNA–ligand interactions, extensive conformational sampling, or non-covalent binding networks within the highly polarized polyanionic ribosomal environment, the integration of empirical dispersion corrections (e.g., Grimme’s DFT-D3 or DFT-D4) or range-separated functionals with built-in dispersion (such as ω-B97X-D) is now a mandatory standard rather than an optional refinement. Without these corrections, standard single-reference DFT methods inherently fail to describe non-covalent attractive forces and π-π stacking, leading to artificial repulsive structures and structurally invalid binding modes. Consequently, contemporary electronic and conformational analyses were performed for lincosamide antibiotics, including clindamycin, lincomycin, and pirlimycin, which bind to the 23S rRNA of the 50S ribosomal subunit at the A- and P-tRNA binding sites and inhibit peptidyltransferase activity [61]. Quantum calculations at the B3LYP/6-31G** level combined with NBO analysis, AIM theory, and NMR parameter calculations demonstrated that the most stable clindamycin conformer contains an intramolecular C=O···H–O hydrogen bond, while charge transfer between the pyrrolidine ring and the methylthiolincosamide sugar moiety constitutes the dominant stabilizing interaction. Crystallographic structures from ribosome-bound clindamycin complexes revealed two distinct conformations of the pyrrolidinyl propyl group within the 23S RNA binding site, while mutations at G2057, A2058, A2059, C2452, and C2611, as well as methylation of A2058, were identified as key determinants of lincosamide resistance. Structural and computational investigations of tetracycline-class antibiotics further demonstrated that tigecycline binds the bacterial 30S ribosomal subunit with substantially greater affinity than classical tetracyclines because of the tert-butyl-glycylamido side chain attached at the C-9 position of the tetracycline scaffold [62,63]. X-ray crystallography showed that tigecycline binds within the primary tetracycline pocket formed by helices h31 and h34 of 16S rRNA and interacts extensively with nucleotide C1054, restricting its conformational mobility and causing a 10–100-fold increase in ribosomal affinity and a 10–30-fold increase in inhibitory activity in translation assays, while simultaneously overcoming major tetracycline-resistance determinants. Additional computational workflows integrating e-pharmacophore modeling, docking, QSAR, and virtual screening identified inhibitors capable of simultaneously targeting streptomycin and paromomycin binding sites within the 30S decoding center. This investigation [64] is focused on the conserved nucleotides A1492, A1493, and G530 of helix 44, whose conformational flipping governs codon–anticodon recognition during translation, while multiple pharmacophore models generated from experimentally resolved ribosome–antibiotic complexes were validated using enrichment factor calculations and decoy datasets. For 50S-targeting oxazolidinones, force-field simulations supervised by DFT and experiment were used because linezolid recognition depends strongly on conformation, hydrogen-bond mechanics, and enantioselectivity [65]. An all-atom ribosomal model of nearly 1600 atoms derived from the *Haloarcula marismortui* 50S structure 3CPW demonstrated that AMBER reproduced the bioactive conformation substantially better than OPLS-AA, with an RMSD of 0.73 Å versus 2.3 Å and avoidance of an artificial minimum of 20 kJ/mol^−1^ above the DFT reference. Even with refined force fields such as AMBER, accurate representation of ribosomal conformational plasticity and induced-fit effects remains incomplete, particularly for highly flexible rRNA domains and transient ligand-induced rearrangements, potentially leading to overestimation of the stability of proposed bioactive conformations [66].

A complementary TD-DFT study of linezolid bound to the *Haloarcula marismortui* 50S ribosomal subunit further demonstrated that the S-enantiomer is the biologically active configuration required for inhibition of protein synthesis [67,68]. Calculations performed at the PBE0/6-311++G(3df,2p) level reproduced UV–Vis and electronic circular dichroism spectra for both free and ribosome-bound linezolid, confirming strong stereoelectronic complementarity between the oxazolidinone scaffold and the 50S PTC environment. Structural reassessment of ribosome-bound antibiotics showed that chloramphenicol binds directly within the A-site crevice of the 50S subunit at the same location as the aminoacyl moiety of A-site tRNA, whereas erythromycin and azithromycin occupy the ribosomal exit tunnel near the PTC through hydrophobic interactions with U2611, A2058, and A2059 [69,70]. Telithromycin preserves the macrolide lactone-ring position, while additionally stacking with U2609 and A752, explaining how mutations or methylation of A2058 lead to MLSBK resistance phenotypes. The same structural analyses demonstrated the feasibility of hybrid antibiotic design through RX-2102, a florfenicol–azithromycin chimaera whose binding to the wild-type *H. marismortui* 50S was confirmed crystallographically (PDB 3OW2). Additional computational analyses of ribosomal druggability identified common structural signatures among 65 experimentally validated antibiotic binding sites extracted from high-resolution ribosome–antibiotic complexes [71]. Binding pockets, defined as all atoms within 6 Å of the ligand, were enriched in non-paired nucleotides, unusual syn-oriented bases, and atypical ribose puckering geometries, leading to the proposal of a druggability index for identifying novel ribosomal antibiotic sites. In the ribosome-preorganization study of the bridged macrobicyclic lincosamide cresomycin [72], DFT was used to define the global minimum-energy conformation of cresomycin, which was subsequently validated by ^1^H-NOESY, single-crystal XRD at 0.84 Å, and ribosome-bound structures. Cresomycin displaced 50% of [^14^C]-erythromycin from immobilized *E. coli* ribosomes at ≤8.2 nM, compared with 35 nM for iboxamycin, while clindamycin exhibited 70-fold lower affinity. In vivo, 25 mg/kg^−1^ qid subcutaneous administration rescued 10/10 mice from LD_90_ cfr-expressing *S. aureus* sepsis, whereas 9/10 vehicle-treated mice died within 2 days. Crystal structures of cresomycin bound to Cfr- and Erm-modified T. thermophilus 70S ribosomes at 2.55 and 2.60 Å revealed only localized structural adaptation, including a 0.6 Å displacement of m^2^m^8^A2503 and a 2.0 Å shift of m^6^2A2058, while the global ligand pose remained essentially unchanged. DFT was also used to support Cu(II) coordination structures of the antituberculosis peptide antibiotic viomycin, while molecular modeling identified RNA-binding sites relevant to ribozyme modulation [73,74]. Viomycin and capreomycin bind in a cleft between the small and large ribosomal subunits by contacting helix 44 of 16S rRNA and helix 69 of 23S rRNA. The study identified two viomycin/ribozyme binding locations, including a deep cavity approximately 10 × 20 Å formed by the P1, P2, and P3 stems, where the Cu(II)–viomycin complex interacts with A14/U15 and G3/U4, while viomycin forms hydrogen bonds with U32 and contacts catalytically important C76 [73,74].

### 5.2. Hybrid QM/MM and FMO Frameworks in Enzyme Active Sites

In enzyme active-site systems, QM/MM and DFT methodologies are essential because classical force fields cannot adequately represent metal–ligand bonds, proton transfer, charge transfer, polarization, or coordination-number changes during catalysis. In subclass B2 monozinc CphA *β*-lactamase from *Aeromonas hydrophila*, SCC-DFTB/CHARMM QM/MM was used because the catalytic zinc occupies the Zn2 site rather than Zn1 and the Michaelis complex cannot be reliably reconstructed through docking alone [75]. The QM region contained 82 atoms, including biapenem, Zn(II), Asp120, Cys221, His263, catalytic water, and His118, while DFT validation in Gaussian 03 at the B3LYP/6-31+G(d) level demonstrated that the substrate binds through the 3-carboxylate oxygen as the fourth Zn ligand and forms a stable hydrogen-bond network maintained during 500 ps MD simulations. Similar SCC-DFTB/CHARMM methodologies were applied to the dizinc L1 metallo-*β*-lactamase from Stenotrophomonas maltophilia, where the 125-atom QM region contained Zn1, Zn2, the bridging hydroxide nucleophile, His116, His118, His196, Asp120, His121, His263, and moxalactam [76]. Zn1 was coordinated by His116, His118, and His196, whereas Zn2 coordinated Asp120, His121, His263, and an apical water molecule, while the hydroxide bridge acted as the nucleophile responsible for *β*-lactam attack. In the mechanistic study of moxalactam deactivation [77], SCC-DFTB/MM free-energy profiles corrected by single-point B3LYP/MM calculations through the GAMESS–CHARMM interface using LANL2DZ effective core potentials for Zn and S and 6-31G(d) for C, O, N, and H increased the calculated nucleophilic-addition barrier from 7.9 to 23.5 kcal/mol^−1^, approaching the experimental value of 18.5 kcal/mol^−1^ derived from k_cat_ = 0.15 s^−1^. The DFT/PCM model identified five stationary points and showed that the dominant TS1 barrier decreases from 27.0 kcal/mol^−1^ in the gas phase to 22.6 kcal/mol^−1^ in water, while mechanistically Zn1 functions as an oxyanion hole, stabilizing the *β*-lactam carbonyl oxygen and Zn2 acts as an electrophilic catalyst, stabilizing the leaving-group nitrogen. Hybrid QM/MM methodologies were also central for investigations of *β*-lactamase-mediated resistance in class A TEM1 *β*-lactamase [78], where the acylation mechanism with benzylpenicillin was modeled using the B3LYP/6-31G+(d)//AM1-CHARMM22 protocol. Ser70, Lys73, Ser130, Asn132, Glu166, and catalytic water were treated quantum mechanically, demonstrating that Glu166 activates the conserved water molecule, which subsequently activates Ser70 for nucleophilic attack on the *β*-lactam carbonyl. Formation of the tetrahedral intermediate represented the highest barrier, with a calculated activation energy of 9 kcal/mol^−1^ closely matching the experimental value of 12 kcal/mol^−1^. Similar QM/MM and DFT investigations of the dinuclear zinc metallo-*β*-lactamase CcrA from *Bacteroides fragilis* [79] showed that Zn1 polarizes the substrate carbonyl group, while Zn2 stabilizes the developing negative charge on the departing amide nitrogen. Zn1 was coordinated by His82, His84, His145, and a bridging hydroxide, whereas Zn2 coordinated Asp86, Cys164, His206, and an apical water molecule. The catalytic mechanism involved simultaneous hydroxide activation by Asp86, substrate polarization by Zn1, and leaving-group stabilization by Zn2, while the proton-transfer step exhibited an activation barrier 1.6 kcal/mol^−1^ higher than nucleophilic attack. Advanced QM/MM electron-density analyses of cephalosporin hydrolysis within the L1 metallo-*β*-lactamase active site demonstrated that a transient N/H–Ow hydrogen bond formed during the transition state governs substrate reactivity [80]. In that case, an additional challenge concerns the accurate computational treatment of transition-metal centers, particularly Zn^2+^ coordination environments, as mechanistic outcomes may vary significantly depending on the selected effective core potentials and treatment of electron correlation, thereby limiting the reproducibility of certain catalytic interpretations.

QTAIM, Fermi-hole analysis, delocalization tensors, source functions, and linear response kernels revealed that reduced localization of the substrate nitrogen lone pair directly correlates with lower catalytic turnover. The catalytic cycle of penicillin acylase from *E. coli* was likewise modeled using QM/MM calculations, demonstrating that the N-terminal *β*Ser1 activates its own hydroxyl nucleophile through an intramolecular proton relay involving its α-amino group rather than a classical Ser–His–Asp catalytic triad [81]. The active-site network composed of *β*Ser1, *β*Ala69, *β*Asn241, *β*Arg263, and *β*Gln23 stabilizes tetrahedral intermediates and maintains catalytic geometry during hydrolysis of penicillin G into 6-aminopenicillanic acid and phenylacetic acid. DFT-supported investigations of aminoglycoside nucleotidyltransferase ANT(2″)-Ia demonstrated that aminoglycosides containing a 2′-NH_2_ substituent exhibit substantially greater catalytic efficiency than 2′-OH analogues in the presence of Mg^2+^, whereas substitution with Mn^2+^ decreases activity approximately tenfold for amino-containing substrates [82]. Tobramycin bound with a dissociation constant of 0.6 μM that improved threefold upon metal–nucleotide coordination, while ATP affinity increased by nearly two orders of magnitude in the presence of divalent metal ions. Modern quantum methodologies also increasingly employ fragment molecular orbital calculations because conventional QM calculations scale prohibitively with biomacromolecular size [83]. FMO calculations implemented in GAMESS and ABINIT-MP divide proteins and nucleic acids into fragments and compute IFIE and PIEDA descriptors, allowing quantitative decomposition of electrostatic, exchange repulsion, charge-transfer, and dispersion interactions while preserving quantum-mechanical treatment of biomolecular systems.

Despite these advantages, applying this standard FMO framework directly to the polyanionic environment of ribosomal networks introduces severe intrinsic complications that are frequently glossed over in empirical workflows. Because conventional fragmentation schemes struggle with the continuously polarized phosphodiester backbone of nucleic acids, dividing a continuous RNA strand into isolated nucleotide fragments requires artificial covalent bond capping. Without deploying advanced polarizable embedding schemes or explicitly calibrated charge-shifting protocols at these arbitrary fragment boundaries, single-point calculations risk generating severe unphysical artifacts in the calculated inter-fragment interaction energies (IFIE) and pair interaction energy decomposition analysis (PIEDA). The accuracy of these DFT and QM/MM calculations strongly depends on the basis set employed, particularly for systems containing transition metals involved in antibiotic coordination and catalysis [84]. Accordingly, the 6-31G and 6-31G* basis sets were extended to third-row atoms K through Zn with polarization functions including Cartesian d-functions for K and Ca and Cartesian f-functions for Sc-Zn, enabling improved treatment of transition-metal coordination geometries and catalytic metal centers encountered in metalloenzymes associated with antibiotic resistance [85].

**Figure 5:**
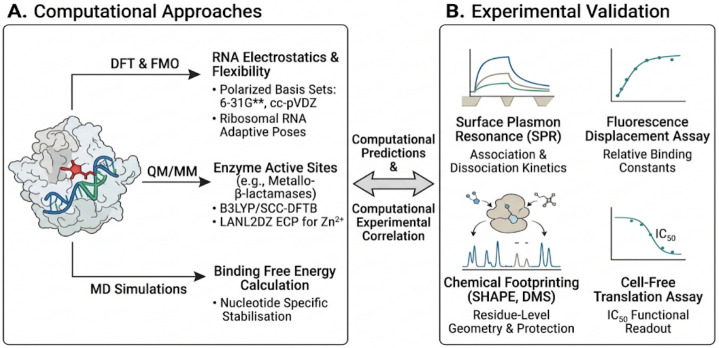
Integrated Computational and Experimental Framework for Ribosomal and Enzymatic Targeting.

Summarizing the collected investigations, for ribosomal systems, the most reliable descriptions are achieved using DFT- and FMO-based approaches combined with polarized basis sets such as 6-31G**, 6-311++G(3df,2p) (where ** indicates polarization functions on both heavy atoms and hydrogen atoms), and FMO-MP2/cc-pVDZ, which effectively reproduce RNA electrostatics, conformational flexibility, and resistance-associated structural adaptation (Figure 5). In enzyme active sites, particularly metallo-*β*-lactamases, hybrid QM/MM methodologies employing B3LYP/6-31+G(d), SCC-DFTB/CHARMM, and LANL2DZ-type effective core potentials for Zn^2+^ centers provide the most accurate representation of catalytic polarization, proton transfer, and substrate activation.

The binding free energies and nucleotide-specific stabilisation energies computed using FMO, MM-GBSA, and TD-DFT approaches for ribosome-targeting antibiotics are supported by experimental evidence [86] from cell-free translation inhibition assays, where IC_50_ values measured in *E. coli* or *T. thermophilus* ribosomal systems provide a direct functional readout of the predicted binding affinities. Fluorescence displacement assays using proflavine or BODIPY-labelled aminoglycoside probes allow quantitative measurement of relative binding constants for novel candidates identified by computational screening, while surface plasmon resonance (SPR) [87] with immobilised 30S or 50S ribosomal subunits provides real-time kinetic association and dissociation rates that can be compared to computationally derived binding free energies. Conformational changes in 16S or 23S rRNA induced by antibiotic binding, predicted through MD-simulated nucleotide displacement and structural adaptation, are experimentally accessible through chemical footprinting (dimethyl sulfate or SHAPE reagents) [88], which identifies protected and reactive nucleotide positions in the presence of the antibiotic, providing residue-level experimental validation of computed binding geometries.

### 5.3. The Critical Gap Between Computational Ideality and Physiological Validation

The transition from localized electronic optimization to in vivo microbiological efficacy represents the most significant failure mode in computational anti-infective design. While multi-scale QM/MM profiles and DFT iterations accurately characterize transition states or coordination architectures under thermodynamic equilibrium, they consistently fail to predict MIC) due to three primary homeostatic and structural barriers:

(a) The Entropic and Dielectric Collapse of Ribosomal Binding Predictions under Macromolecular Crowding. In computational screenings targeting the 30S decoding center or the 50S peptidyl transferase center (PTC), single-point free-energy calculations (such as FMO-MP2 or MM-GBSA) routinely yield highly exergonic binding energies (ΔG_bind_ from −12 to −18 kcal/mol) for highly charged structures like aminoglycoside derivatives or oxazolidinones. However, in the physiological environment of the bacterial cytoplasm—where macromolecular crowding occupies up to 30–40% of the total volume—the local dielectric constant fluctuates unpredictably between the bulk water baseline (ε ~ 78) and restricted interfacial boundaries (ε ~ 4–10).

The Specific Failure Mode: Truncated active-site DFT models completely freeze out the long-range electrostatic screening and dynamic target reorganization mediated by highly mobile Mg^2+^ solvation shells. Quantitatively, omitting the entropic penalty of displacing tightly bound water molecules and counterions causes computational models to overestimate structural stability, leading to a systematic discrepancy of 3 to 7 kcal/mol against experimental isothermal titration calorimetry (ITC) data [89]. Consequently, virtual screens generate high false-positive rates, prioritizing noncanonical binders that fail entirely in cell-free translation assays.

(b) Active Efflux Kinetics vs. Frontier Orbital Softness Descriptors. In the coordination chemistry of fluoroquinolones (e.g., ofloxacin or ciprofloxacin complexes), DFT models emphasize that a reduction in the HOMO–LUMO gap (ΔE dropping from 3.83 eV to 1.77–2.44 eV) directly translates into enhanced chemical softness and localized reactivity. The implicit assumption is that this electronic activation guarantees superior bactericidal potency.

The Specific Failure Mode: In vivo drug accumulation is not an equilibrium phenomenon dictated by orbital alignment, but a kinetic competition between passive outer-membrane permeability and active extrusion. For instance, despite optimal calculated binding free energies or enhanced electronic softness, transition-metal complexation significantly alters the molecule’s global lipophilicity and spatial volume. In Gram-negative pathogens like Pseudomonas aeruginosa, these structural modifications render the complex an ideal substrate for multi-component, proton-motive-force-driven efflux networks such as the MexAB-OprM or AcrAB-TolC pumps. Because these pumps continuously translocate the drug out of the periplasm at rates out-competing passive influx, the systemic concentration never reaches the thermodynamic threshold required to occupy the target, resulting in a 4- to 16-fold increase in the actual MIC despite superior computational descriptors [90,91].

(c) *Metallo-Regulation and The Femtomolar Trap in Coordination Therapeutics.* Computational designs of mixed-ligand antibiotic complexes (e.g., thiamphenicol, imipenem, or sulfamethoxazole bound to Cu^2+^, Zn^2+^, or Fe^3+^) frequently demonstrate spontaneous, highly exergonic chelation energies (ΔG_chel_ < −20 kcal/mo in gas-phase or PCM models), assuming the complex will remain intact during transport to the biological target.

The Specific Failure Mode: This paradigm ignores the strict thermodynamics of bacterial transition-metal homeostasis. In the cellular matrix, free transition metal ions do not exist in a bulk solution state; they are tightly sequestered by ultra-high-affinity metallochaperones and sensory proteins (e.g., Zur, Fur, or CueR) down to femtomolar (10^−15^ M) or attomolar levels. When a metal–antibiotic complex enters the cell, the massive thermodynamic gradient imposed by empty, high-affinity intracellular binding sites causes immediate competitive ligand exchange. The coordinated metal ion is stripped from the antibiotic scaffold before it can interact with the target enzyme or ribosome, rendering the computational stability profiles irrelevant and reducing the in vivo efficacy to that of the uncomplexed parent drug.

## 6. Conclusions

Antibiotic action cannot be fully understood from molecular structure alone, as it must be interpreted in terms of the electronic events that determine recognition, activation, and resistance. For *β*-lactamase-mediated hydrolysis, hybrid QM/MM methodologies provide the most accurate mechanistic description, particularly when combined with B3LYP or M06-2X functionals and polarized split-valence basis sets such as 6-31G(d), 6-31+G(d), or 6-311+G(d,p). In metallo-*β*-lactamases, effective core potentials such as LANL2DZ remain essential for reliable treatment of Zn^2+^ catalytic centers, while high-level DLPNO-CCSD(T)/CBS corrections significantly improve transition-state energetics and agreement with experimental kinetics.

In antibiotic–metal complexes, B3LYP combined with 6-311G(d,p), 6-311+G(d,p), or def2-SV(P), together with LANL2DZ-type pseudopotentials and PCM/CPCM solvation models, provides a balanced framework for describing coordination geometry, HOMO–LUMO redistribution, and biologically relevant electronic effects. These calculations consistently show that coordination through β-lactam carbonyl, carboxylate, quinolone keto, and nitrogen donor sites enhances electronic softness, charge delocalization, and antimicrobial activity.

For ribosomal systems, multiscale strategies integrating docking, MD, MM-GBSA, DFT, TD-DFT, and FMO calculations must strictly reject outdated non-dispersive approximations, as no isolated uncorrected quantum approach can faithfully reproduce the non-covalent landscape of large RNA–protein assemblies. While functionals such as B3LYP and PBE0 are widely deployed alongside polarized basis sets (e.g., 6-31G, 6-311++G(3df,2p)), this review establishes that for all non-covalent binding complexes, RNA–ligand networks, and conformational sampling, the application of Grimme’s dispersion (DFT-D3/D4) or dispersion-encoded hybrid functionals is a mandatory prerequisite to prevent catastrophic delocalization errors and structural artifacts. Functionals such as B3LYP and PBE0 combined with polarized basis sets including 6-31G**, 6-311++G(3df,2p), and FMO-MP2/cc-pVDZ are particularly effective for analyzing stereoelectronic complementarity, nucleotide-specific stabilization, and resistance-associated conformational adaptation within the 30S and 50S ribosomal subunits. A comparative methodological assessment of the principal quantum–chemical strategies discussed throughout this review is presented in Table 2.

The investigations indicate that the future of antibiotic research depends on integrating quantum chemistry with dynamic biological modeling and experimental validation. For β-lactamase inhibitors, future studies should prioritize transition-state analogues that disrupt proton-transfer networks and Zn^2+^-assisted polarization rather than merely occupying the active site. Research on metal-based antibiotics should focus on rational selection of metal ions, donor atoms, redox behavior, and solvation-stable coordination geometries to maximize antimicrobial activity while minimizing nonspecific toxicity. For ribosome-targeting antibiotics, calculations of conformational preorganization, RNA electrostatic complementarity, and resistance-site accommodation should become central design principles. Overall, the investigations reviewed here indicate that the future of antibiotic research will increasingly depend on the integration of quantum chemistry with dynamic biological modeling and rigorous experimental validation. DFT and QM/MM methodologies provide valuable mechanistic insight into antibiotic reactivity, target recognition, and resistance mechanisms, but their predictive power remains subject to methodological limitations and the inherent complexity of biological systems. Rather than serving as standalone predictive platforms, these approaches are most effective when used to generate testable mechanistic hypotheses, prioritize promising molecular systems, and support subsequent biochemical, microbiological, and pharmacological investigations. Continued progress in antibiotic discovery will therefore require close integration of quantum–chemical modeling with experimental pharmacology, structural biology, and systems-level studies of antimicrobial resistance.

## Figures and Tables

**Table 1:** Boolean query strings deployed during the initial retrieval phase.

Core Target Domain	Boolean Query Strings
Enzymatic Degradation Mechanisms	(“DFT” OR “quantum chemical”) AND “antibiotic calculations” AND (“beta-lactamase” OR “hydrolysis”)
Metallo-β-lactamase Catalysis	(“QM/MM” OR “hybrid quantum mechanics”) AND “enzyme active sites” AND (“NDM-1” OR “zinc coordination”)
Ribosomal Target Recognition	(“fragment molecular orbital” OR “FMO” OR “TD-DFT”) AND “ribosome” AND (“antibiotic binding” OR “decoding center”)
Coordination Chemotherapeutics	(“DFT” OR “density functional theory”) AND “antibiotic-metal interactions” AND (“HOMO-LUMO gap” OR “basis set preference”)

**Table 2:** Comparative evaluation of predictive potential and methodological performance of quantum–chemical approaches in antibiotic research.

Research Object	Preferred Computational Strategy	Recommended Functional/Basis Set/ECP	Advantages	Limitations
*β*-Lactam ring hydrolysis	Hybrid QM/MM + enhanced sampling + ONIOM refinement	B3LYP or M06-2X with 6-31G(d), 6-31+G(d), 6-311+G(d,p); LANL2DZ for Zn^2+^; DLPNO-CCSD(T)/CBS corrections.	Accurate description of proton-transfer pathways, transition states, oxyanion stabilization, and catalytic Zn^2+^ polarization; strongest correlation with experimental kinetics	Inherent lack of long-range dispersion; completely unreliable for π-π stacking and non-covalent interactions unless explicitly corrected via DFT-D3/D4 methodologies. High self-interaction error.
Antibiotic–metal complexes	DFT + TD-DFT + implicit solvation	B3LYP with 6-311G(d,p), 6-311+G(d,p), def2-SV(P); LANL2DZ; PCM/CPCM.	Reliable prediction of coordination geometry, HOMO–LUMO redistribution, donor-atom selectivity, and spectroscopic behavior	Biological activity cannot be predicted solely from HOMO–LUMO descriptors; transition-metal treatment remains functional-dependent.
Ribosomal subunits and active sites	Multiscale docking + MD + MM-GBSA + TD-DFT + FMO	B3LYP, PBE0; 6-31G**, 6-311++G(3df,2p); FMO-MP2/cc-pVDZ.	Effective for describing RNA electrostatics, conformational adaptation, and nucleotide-specific stabilization	Inherent failure of standard functionals (e.g., B3LYP) to describe non-covalent binding, conformational sampling, and RNA–ligand interactions unless explicitly modified via mandatory dispersion corrections (DFT-D3/D4); catastrophic structural distortion in uncorrected models.

## Data Availability

The authors confirm that the data supporting the findings of this study are available within the article.
